# Experience of an Italian Pediatric Third Level Emergency Department during the 2022–2023 Bronchiolitis Epidemic: A Focus on Discharged Patients and Revisits

**DOI:** 10.3390/children11030268

**Published:** 2024-02-21

**Authors:** Giovanna Iudica, Daniele Franzone, Marta Ferretti, Barbara Tubino, Stefania Santaniello, Giacomo Brisca, Clelia Formigoni, Erica Data, Emanuela Piccotti

**Affiliations:** 1 Department of Health Sciences (DISSAL), University of Genoa, 16132 Genoa, Italy; s3841103@studenti.unige.it; 2 Department of Neuroscience, Rehabilitation, Ophthalmology, Genetics, Maternal and Child Health (DINOGMI), University of Genoa, 16132 Genoa, Italy; s3805091@studenti.unige.it (C.F.); s5121690@studenti.unige.it (E.D.); 3 Pediatric Emergency Room and Emergency Medicine Unit, IRCCS Giannina Gaslini Institute, 16147 Genoa, Italy; martaferretti@gaslini.org (M.F.); barbaratubino@gaslini.org (B.T.); stefaniasantaniello@gaslini.org (S.S.); emanuelapiccotti@gaslini.org (E.P.); 4 Intermediate Care Unit, Emergency Department, IRCCS Istituto Giannina Gaslini, 16147 Genoa, Italy; giacomobrisca@gaslini.org

**Keywords:** pediatric emergency medicine, pediatric emergency department, bronchiolitis, RSV, SARS-CoV-2, children

## Abstract

The aim of this study was to describe the 2022–2023 bronchiolitis epidemic season (the second after COVID-19 pandemic and the first without social restriction), focusing on patients discharged home from a pediatric emergency department (PED) and on those revisited within 72 h. We performed a retrospective observational study in an Italian tertiary care children’s hospital, reviewing PED accesses from 1 October 2022 to 31 March 2023. The number of hospitalizations for bronchiolitis was extracted from hospital discharge forms. A total of 512 patients diagnosed with bronchiolitis were admitted to PED (2.8% of total admissions). Accesses increased sharply from November to January, with a peak in December, in both admissions and hospitalizations. More than half of the patients (55.5%) were safely discharged home, while 38 (13.4%) came back to PED for a revisit. Overall PED accesses and hospitalizations for bronchiolitis increased since the previous epidemic season, and particularly compared to the pandemic and pre-pandemic eras. Empowering the collaboration between all healthcare provisioners is fundamental to suitable management of patients. Monitoring the epidemiology and seasonality of bronchiolitis is a starting point for an effective internal organization of pediatric departments and to further evaluate its socio-economic burden.

## 1. Introduction

Bronchiolitis is an acute viral infection that most affects the lower respiratory tract of children under the age of twelve months, representing a major cause of pediatric emergency department (PED) visits and hospitalization in individuals of this age, leading to a substantial socioeconomic burden on health-care services and communities worldwide [1,2]. High mortality rates are described in developing and industrialized countries as well, and the disease represents the main cause of death due to viral infection during the first year of life [3]. 

Clinical presentation of children with acute bronchiolitis widely ranges from mild to severe respiratory distress, potentially resulting in respiratory failure [1]. More serious clinical patterns are generally associated with the presence of risk factors (e.g., prematurity, congenital anomalies, immunodeficiency, chronic lung disease) [4]. Despite the high prevalence of the disease, it is a clinical diagnosis based on medical history and physical examinations searching for rhinorrhea, crackles, wheezing, dyspnea, use of accessory respiratory muscles, skin colour changes, feeding difficulties, lethargy and fever [4]. There is a slight annual variability in the characteristics of the disease that alternates according to geographical region and/or the seasons. In Europe, the period accounting for the highest number of diagnoses is from October to April, with a peak varying from December to February [5].

Respiratory syncytial virus (RSV) is the most prevalent infective agent, with the highest burden being in the first three months of life [6,7]. Other possible causative agents (alone or as a co-infection) are Rhinovirus (RV), Parainfluenza virus, Metapneumovirus (MPV), Influenza virus, and Adenovirus [1,2,3,4,5,6,7,8]. Although more than 60% of children contract RSV by the age of one year, and 90% by two years [9], nearly 20% of patients necessitate pediatric practitioner visits or emergency department evaluation due to respiratory symptoms, with a minority (2–4%) who need hospitalization [10].

During the COVID-19 pandemic, the circulation of many respiratory viruses changed, including pathogens responsible for bronchiolitis. An overall reduction in influenza and other respiratory infections was observed at that time, influenced by the adoption of restrictive (lockdown and social distancing) and preventive measures (use of face masks and increased attention to hand hygiene) to fight the spread of SARS-CoV-2 [11]. As a consequence, a reduction in the number of PED accesses and hospitalizations for common viral infections was observed [12,13]. After the introduction of mass vaccination campaigns in 2021, a general loosening of social restrictions occurred. A recrudescence in common viral infections was observed, with a particular increase in bronchiolitis cases during the season of 2021–2022, albeit characterized by partial restrictive measures, such as use of mask in closed environment and fiduciary isolation for people affected by COVID-19 [11,14]. During 2022, a further general loosening of the last social restrictions occurred due the higher percentage of people vaccinated for SARS-CoV-2 and the circulation weakened variants of the virus causing less-severe disease.

The aim of this study is to retrospectively describe the 2022–2023 bronchiolitis epidemic season, the second after the COVID-19 pandemic and the first without social restriction, in the context of a tertiary care Pediatric Emergency Department (PED) to describe the epidemiology and the priority and urgency codes of patients accessing ED for bronchiolitis. Moreover, we focus on patients discharged at home and, among this cohort, patients revisited on a second access within the first 72 h (PED revisits) in order to describe clinical characteristics and, when available, to provide an etiological assessment.

## 2. Materials and Methods

This monocentric retrospective observational study was performed in an Italian pediatric referral acute care centre, IRCCS Istituto Giannina Gaslini, located in Genoa, in the northwest of Italy.

The hospital is provided with a third level PED that carries an average admission rate of 35,000 patients per year.

PED triage is performed by a trained nurse and involves the identification of a priority code assigned to the patient based on clinical condition, escalation risk and availability of resources. The coding system consists of five numeric priority codes (from 1, the most serious, to 5, the least serious), each associated with a colour code (1 red, 2 orange, 3 blue, 4 green, 5 white). Notably, 1 constitutes an emergency, 2 and 3 refer to an urgency, 4 and 5 indicate non-urgent care. The same code scheme is used to identify a severity code at discharge after medical assessment.

We collected overall PED accesses and admissions for bronchiolitis referring to the period October–March of the 2017–2018, 2018–2019, 2019–2020, 2020–2021, 2021–2022 and 2022–2023 epidemic seasons.

We reviewed electronic charts of all children admitted with a diagnosis of “acute bronchiolitis”, classified with 466.11 and 466.19 codes of the International Classification of Diseases 9th edition (ICD-9-CM) from 1 October 2022 to 31 March 2023.

The diagnosis of bronchiolitis was made through clinical assessment undertaken by the physician on the basis of signs and symptoms characterizing the disease (e.g., rhinorrhea, cough, wheeze or crackles during chest auscultation, dyspnea, feeding difficulties, lethargy, fever). 

Amount, priority code assigned at triage, and outcome (discharged home, hospitalized, brief intensive observation-OBI, outpatient clinic, patient leaves before visit, patient refuses hospitalization or transferred to another hospital) were analyzed. 

OBI is a holding unit dedicated to the clinical stabilization of patients who, on preliminary medical appraisal, are estimated not to require a hospital stay of more than 24 h.

In cases of bronchiolitis diagnosis and patients discharged home or PED revisits, severity code assigned by medical evaluation and other demographic and clinical data (age, sex, gestational age, siblings, parents’ nationality, comorbidities, oxygen saturation, respiratory rate-RR, therapy given in PED, therapeutic instructions at discharge from PED) were also collected. Identification of the causal agent and viral agent was performed using a nasopharyngeal swab with a multiplex polymerase chain reaction (PCR) assay and, after the introduction in late December 2022 in the PED, with a rapid antigenic test for RSV on fluorescent immunoassay, performed with a STANDARD F2400 SD Biosensor INC by SD Biosensor (C-4&5 Floor, 16, Deogyeong-daero 1556beon-gil, Yeongtong-gu, Suwon-si, Gyeonggi-do, 16690, Republic of Korea). Furthermore, upon admission to the PED, patients with respiratory symptoms were routinely tested with a rapid antigen swab for SARS-CoV-2.

The number of hospitalizations for bronchiolitis was extracted from hospital discharge forms for the period October–March of the 2017–2018, 2018–2019, 2019–2020, 2020–2021, 2021–2022 and 2022–2023 epidemic seasons. The hospital discharge form is an ordinary tool for collecting information on every patient discharged from all public and private healthcare centres nationwide.

Descriptive statistics were performed. The median and range are presented for nonnormally distributed variables, numbers and rates for categorical variables. Normality has been verified using the Shapiro—Wilk test.

Due to the observational and retrospective nature of the study conducted on routinely collected and anonymous data, the study did not require ethical approval. In this hospital, however, consent to anonymous use of clinical data for research and epidemiological purposes is routinely requested upon admission or diagnostic procedures.

## 3. Results

### 3.1. Gaslini PED and Bronchiolitis

During the period under review (October 2022–March 2023), a total of 512 patients were admitted to PED and received an ICD-9 code for bronchiolitis (2.8% of total accesses), distributed monthly as follows: 15 (2.9%) in October, 113 (22.1%) in November, 181 (35.4%) in December, 106 (20.7%) in January, 56 (10.9%) in February and 41 (8%) in March. In the months of November–January, there was a sharp increase in the number of accesses for the disease, with a peak in December.

**Figure 1:**
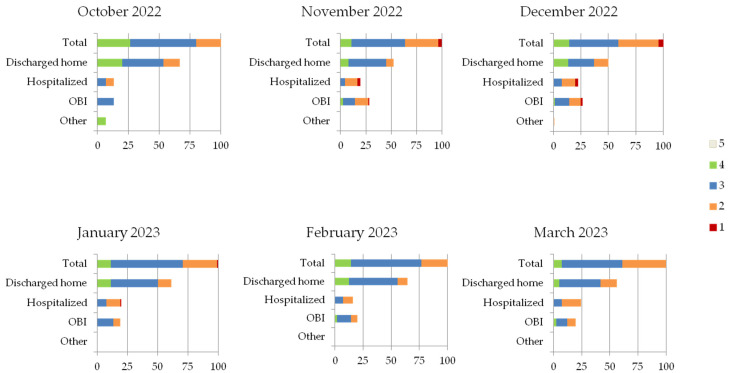
Proportion of PED accesses for bronchiolitis, subdivided monthly by priority code established at triage and by outcome. Other (patient refuses admission or leaves before the visit), as a category, has also been noted.

In Figure 1, PED accesses for bronchiolitis are described monthly, subdivided by outcome and by the priority code established at triage. More details about distribution of PED accesses, priority codes and outcome after PED visit are provided in the Supplementary Materials.

The most frequently represented priority codes were those of medium-high priority (codes 2 and 3) for all months, except October, when codes of medium-low priority (codes 3 and 4) were assigned. More serious cases (code 1) were only seen from November to January (13 cases or 2.5% of the overall total). No patients were labelled with a code 5 at PED triage.

With reference to the entire period under study, clinical conditions of the patients permitted the clinicians to safely discharge them home in more than half of cases (55.5%), allowing for management of the pathology in collaboration with the community services. 

A period of observation in OBI was employed in 23.6% of cases, especially from November onwards, to the extent of about more than 19% of total accesses and typically for medium-high priority codes (2 and 3). 

Among patients diagnosed with bronchiolitis in the emergency department, 20.5% were hospitalized.

The months with the greatest pressure on PED (November 2022–January 2023) saw an increase in pathology hospitalizations, which were 22, 41, 21 in November 2022, December 2022 and January 2023, respectively. December saw a peak in both admissions and hospitalizations. In addition, one patient refused admission and another one left before being visited (these outcomes are identified as “other” in Figure 1).

Furthermore, overall hospitalizations for bronchiolitis during October 2022–March 2023 reached 265, showing an increase, as those occurring during the same period in the previous epidemic season only amounted to 244. There is indeed evidence of a recent consistent rise in hospitalizations for bronchiolitis when also compared to the pandemic and pre-pandemic eras, where hospitalizations never exceeded 200 and were small in number, especially in the 2020–2021 epidemic season. 

**Figure 2:**
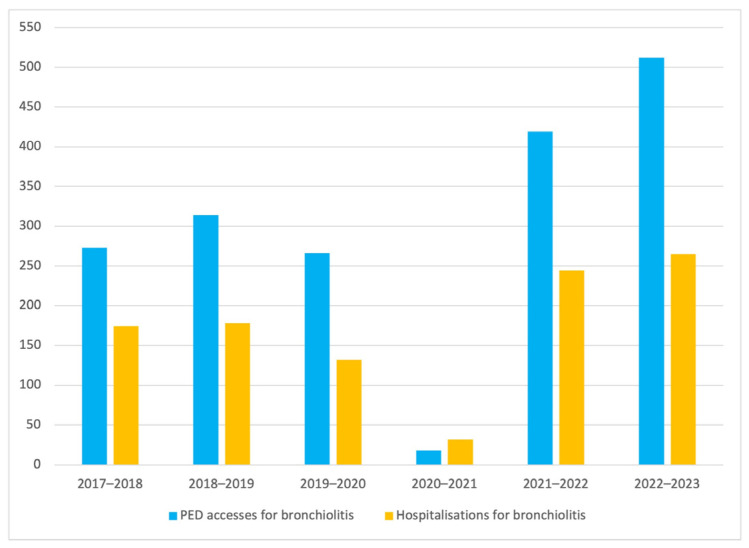
PED accesses and hospitalizations for bronchiolitis during epidemic seasons examined (2017–2023).

Examination of the epidemic seasons from 2017 to 2023 also showed an increase in PED accesses for bronchiolitis, especially after the drastic decline observed during 2020–2021, and even when compared to the era before COVID-19 pandemic. Figure 2 illustrates PED accesses and hospitalizations for bronchiolitis in the different epidemic seasons examined.

In Table 1, the total PED accesses, admissions and hospitalizations for bronchiolitis are described.

### 3.2. Patients Discharged Home

More than half of the patients (55.5%) diagnosed with bronchiolitis were discharged home. Demographic and clinical characteristics of these patients are described in Table 2. All were aged ≤ 12 months, and most of them were males (61.6%). The majority (91.8%) were born at term, while 15.5% presented risk factors. Over half of them had Italian parents and at least one sibling. A causal viral agent test was performed in 64 patients: 24 (37.5%) tested positive for RSV and 40 (62.5%) negative. During their PED stay, all patients performed a rapid antigenic test for SARS-CoV-2, resulted negative in all the cases, and no one presented with respiratory failure (i.e., SpO_2_ < 90%).

Most of these patients were assigned a triage priority code of 3 (59.8%), followed by 4 (20.1%) and 2 (20.1%). At discharge, almost all patients were classified with a severity code of 4 (96.8%) and the remainder with code 3 (3.2%). No patients were categorized with code 1 or 5 either upon admission nor on discharge from PED.

In regard to treatment in PED, the most commonly used medications were bronchodilators: adrenaline in association with ipatropium bromide was prescribed in 52.5% of individuals, while adrenaline alone was used in 19.3% of patients. None of the patients were prescribed β2-agonists. None required supplementary oxygen administration.

At discharge, adrenaline therapy was recommended in almost all cases (96.5%). Supportive care (i.e., nasal suctioning, nasal irrigation using isotonic 0.9% sodium chloride and fractionated and frequent meals) was also always recommended, as well as ongoing follow-ups with the Primary-Care Physician (PCP).

### 3.3. PED Revisits

Among the 284 patients discharged home, 38 (13.4%) came back to PED within 72 h for a second clinical evaluation (PED revisits). One patient left before medical examination. The available demographic and clinical characteristics of patients revisited are detailed in Table 3. 

All individuals were ≤10 months old (84.2% aged ≤ 6 months), and the frequency of revisits was the same in both sexes. Almost all of these patients were born at term (92.1%) and had no risk factors (97.4%). Also, 60.5% of them had Italian parents and at least one sibling. 

Etiological assessment (in the PED or, for patients admitted, during hospitalization) has been performed in 21 patients: 15 (71.4%) tested positive for RSV and 6 (28.6%) negative. Among these patients, 12 of them were tested with a multiplex PCR via nasopharyngeal swab: 3 of 9 patients RSV positive presented a co-infection with another respiratory causative agent (1 RSV and metapneumovirus, 1 RSV and rhinovirus, 1 RSV and haemophilus influenzae). Furthermore, among 3 RSV-negative patients, one resulted in a positive for metapneumovirus. All patients tested negative for SARS-CoV-2.

Upon the first access to PED, no one presented respiratory failure. The priority codes assigned were mainly 3 (60.5%) and 2 (26.3%), and finally 4 (10.5%) and 1 (2.6%). The severity code defined at the medical evaluation was 4 in 76.3% of cases, followed by 2 (13.2%) and 3 (10.5%). In the first episode, none of the patients were admitted to a pediatric ward; the majority of them were discharged home immediately (76.3%), whereas 23.7% of them underwent a brief period of surveillance in OBI. 

On the other hand, on the occasion of the PED revisits, home discharge was decided for 12 patients (31.6%), whereas 12 patients (31.6%) were observed in OBI, half of whom were discharged home and the remainder admitted. Furthermore, 13 patients (34.2%) were admitted to a pediatric ward for subsequent treatment. The total number of patients hospitalized was 19 (50%). 

During the PED revisits, one patient presented respiratory failure. If the rating of priority codes could be considered to be distributed with the same proportions as the first PED access, the same cannot be said for the grading of severity. In fact, the medical assessment assigned a code of 3 and 2 to a higher number of cases, amounting to 47.4% and 18.4%, respectively, whereas only 31.4% of individuals were identified with code 4.

The most commonly used medications were bronchodilators, mainly adrenaline in association with ipratropium bromide (88.2% and 90.9% of cases at first access and revisit, respectively). No patients were given β2-agonists. Supplementary oxygen administration was required during one revisit.

Upon discharge from PED, adrenaline was prescribed in almost all cases (≥94,7%) at both first and second access.

## 4. Discussion

The aim of the study was to retrospectively describe the 2022–2023 bronchiolitis epidemic season by collecting information on all children younger than 24 months accessing our third level PED for this pathology in order to evaluate the seasonality of the disease. The secondary aim was to focus on patients managed in the ED and safely discharged home, with a particular regard for those who made a second access to PED within 72 h from discharge. 

A general anticipation in the starting of the season and an anticipated peak were noted with a reduced duration of the season from the pre-pandemic period. The SARS-CoV-2 pandemic period was characterized by a dramatic decrease in viral infections in the pediatric population with a notable reduction in PED accesses [15]. Lockdown measures and social distancing strategies aiming to contain the SARS-CoV-2 spread had a collateral effect on other respiratory viruses that, with SARS-CoV-2, share the way of spreading via droplets from infected people coughing, sneezing or talking. 

With the introduction of effective and safe SARS-CoV-2 vaccines and the reduction in social and preventive restrictions, the subsequent 2021 season saw an abnormal bronchiolitis outbreak in terms of overall incidence, as well as anticipation of the usual peak and a shortened duration of the epidemic season reported in Italy [14,16] and all over the world [17,18,19]. That epidemiological season was, however, characterized by partial social restrictive measures, although milder than the year before, such as social distancing, use of facial masks in closed environments, limited number of people at social events and fiduciary isolation for people affected by COVID-19. 

A sharp decrease in bronchiolitis cases at the end of December 2021 and the ending of the RSV season by January 2022, concurrent with a sudden increase in SARS-CoV-2 pediatric cases, has been noted, although it was unexpected [20]. These factors can be explained based on the theory of the viral interference phenomenon: it has been proven that respiratory viral infection could prevent the super-infection of other respiratory pathogens due to the activation of the innate immunity that confers to respiratory mucosal cells the ability to counteract a second virus replication [21,22,23]. An opposite frequency in hospital admission for RSV and SARS-CoV-2 confirmed a relationship between the two infective agents [16]. Another hypothesis raised is about the “immunological debt”: the comeback of a virus in a community that has not encountered it prior, and so has no antibodies against it, leading to an increased transmission, a more elevated number of children presenting the disease and a more severe course. Moreover, children born during the SARS-CoV-2 pandemic may have received less immunological stimulation during their first year of life due to social restrictions, leading to more cases of bronchiolitis in the subsequent season [11,14]. 

The 2022–2023 season, the first without real restrictions in social behaviors in Italy (the ending of the state of emergency nationwide was declared on 31 March 2022), confirmed the previous year’s trend of a shorter and anticipated bronchiolitis season starting in October 2022, with an anticipated peak between November and December 2022, a reduction in the number of cases starting in January towards March 2023. 

This is a different distribution of cases compared with the pre-pandemic “bronchiolitis season”, ranging from November to April, with a peak in February [24]. Data from our PED showed that the overall number of accesses for bronchiolitis increased not only since the pandemic era but also compared with the pre-pandemic period, particularly in terms of the number of cases and the percentage on total accesses. Moreover, the total number of hospitalizations for bronchiolitis increased since the previous epidemic season and compared to the pandemic and pre-pandemic eras. The number of hospitalizations for bronchiolitis has been extracted from electronic chart data. During the season of 2020–2021, a more elevated number of patients hospitalized for bronchiolitis than the total number of patients accessing PED for this condition has been noted: during that season, characterized by a reduced circulation of RSV, newborns and toddlers hospitalized for SARS-CoV-2 infection and clinical monitoring of the condition may have developed symptoms consistent with bronchiolitis during their hospitalization period. Moreover, a mild overestimation in the number of hospitalizations for bronchiolitis may have occurred.

The upper age cut-off of the study was set to two years of age, firstly due to the lack of a general consensus about [1,4,25] and in order not to exclude children diagnosed with bronchiolitis in their second year of life who could have missed the infection during their first [26]. It is notable that nearly 98% of patients on the first access and all the patients revisited were younger than one year. More than 90% of the patients were born full term, 60% had at least one sibling, while nearly 10% presented a risk factor for a serious clinical pattern of disease [4]. More than half of the patients evaluated in PED for bronchiolitis were safely discharged with an indication of home management in collaboration with PCPs. The rate of discharged patients was in line with records observed in other Italian hospitals [14,27]. According to the latest American Academy of Pediatrics guidelines, the pulse oxygen saturation (SpO2) threshold of 90% has been established as one of the criteria for hospitalization [4,5,6,7,8,9,10,11,12,13,14,15,16,17,18,19,20,21,22,23,24,25,26,27,28], although no clear recommendation for an observation period in the emergency department is available for patients presenting with SpO2 between 90–92%. Among the discharged patients, none of them were admitted in critical condition or presenting acute respiratory failure, while the minimum SpO2 measured was 93% at triage. In our PED, a period of brief intensity observation (OBI) is often reserved with patients not in critical conditions but presenting with SpO2 between 90% and 92% who are not in a condition to allow a safe discharge. In our center, during the epidemiological season under review, nearly 24% of the accesses for bronchiolitis were recovered in OBI for a brief period of observation. Due to the nature of the study, clinical characteristics of the patients recovered in OBI have been excluded. Furthermore, a percentage of these patients has been recovered in a pediatric ward for worsening of clinical conditions.

A certain quote of respiratory distress was observed in all the patients evaluated, while median respiratory rate values were normal for age [29], although some patients presented with tachypnea. As a compensatory mechanism for inadequate ventilation, newborns and infants are dependent on an increased respiratory rate and distress due to their chest anatomy [30]. During the first months of the season 2022–2023, etiological assessments of patients discharged for bronchiolitis were very limited because only multiplex PCR on a rino-pharyngeal swab was available (only one patient tested and resulted positive for RSV). Since the introduction in late December of the rapid antigenic test, the number of patients tested for RSV increased. One out of three patients tested and discharged from PED resulted in a positive for RSV. This is undoubtedly a useful tool for the correct management of patients affected by bronchiolitis, even in the context of the PED. Furthermore, all the patients undertook a SARS-CoV-2 antigenic test that resulted negatively. Dealing with patients revisited, more than half of them received at least an antigenic test for RSV during their accesses in PED. Moreover, nearly all the patients recovered received a multiplex PCR during their admission.

In addition to general care, the majority of the patients were treated with bronchodilators, in accordance with previously established internal protocol for bronchiolitis treatment. Patients that did not receive bronchodilators in the emergency department were already undergoing therapy with them, as prescribed by general pediatricians, or in good conditions not necessitating it.

Predicting a safe discharge is not easy due to the characteristics of bronchiolitis itself and a population of patients almost entirely constituted by newborns and infants. A management algorithm for patients affected by bronchiolitis accessing emergency department has been proposed trying to identify which factors were associated with a safe discharge at home (age, feeding ability, clinical and ventilatory parameters, i.e., SpO2 at triage, respiratory rate, respiratory distress, need of supplementary oxygen) [31,32]. Further investigations are needed to determine factors really predictive of a safe discharge from ED.

In our setting of a high number of children discharged from PED, the collaboration between hospital and PCPs is fundamental in terms of screening patients to reduce inappropriate accesses. Their role is even more important during epidemic season when hospital pressure is higher: office visit or home clinical evaluation, close monitoring of children discharged from hospital and the availability of daily consultations may reduce hospital overcrowding. On the other hand, general pediatricians have the important role of recognizing patients discharged from ED who may need a second access for hospitalization due to the evolving characteristics of the pathology, or the identifications of factors associated with a poor outcome at home. Among patients who accessed PED within 72 h, an increased necessity of hospitalization was noted: only a minority of them had been discharged home with a reinforcement to follow-up with general pediatrician, and nearly two-thirds were admitted for clinical monitoring in OBI or a pediatric ward. In a minority of cases oxygen supplementation or non-invasive ventilation with HFNC was needed during the hospitalization.

Bronchiolitis in children determines a substantial load on health-care services, requiring economic investments and seasonal planning in terms of human resources, provision of medicines and supplies for pediatric care [33]. Considering the significant RSV disease burden, it is fundamental to assess the economic impact on the limited healthcare resources (which must be allocated cleverly) [34]. High hospitalization costs have recently been described for bronchiolitis in a tertiary care hospital in Italy [35], especially in the case of RSV etiology, associated with longer hospitalization and more frequent admissions to the pediatric intensive care unit (PICU) [36,37,38]. Costs are augmented for PICU hospitalization and longer lengths of stay in the pediatric ward. According to a previous European study, the average costs for a patient younger than 12 months hospitalized in PICU for bronchiolitis were more than four times higher than for low intensity wards and over 20 times higher than for patients managed in the PED and discharged [39].

In an epidemic setting of high ED pressure and hospitalizations, a worsening factor could be the reduced health literacy among the general population [40]. Low health literacy can affect parents’ acquisition of basic health information and knowledge about children’s health outcomes concerning disease prevention and care [40,41], with an impact on the ability to understand a child’s diagnosis and treatment instructions [42]. Among all the consequences to the healthcare service, higher ED use is the main issue affecting hospital pressure, and it is exacerbated during epidemic season [43]. In a USA setting, nearly one-third of parents of children presenting to ED have low health literacy, thus determining a terrific increment in terms of costs for healthcare [43]. A minority status and a lower level of education has been associated with a more elevated trend in seeking care in the ED, particularly for non-urgent reasons [44]. A common misperception about PCPs’ availability and capability influences PED access for low acuity diseases, often presented due to parents’ perception of the higher severity of children’s conditions than perceived by medical staff [45].

Dealing with these knowledge problems, an instrument that may also reinforce the alliance between ED healthcare provisioners and PCPs is the ability to give information to patients’ caregivers using clear communications, adapting language to the family level of education and offering developmentally appropriate instructions to children [42]. It is the responsibility of the pediatricians to do all they can to identify gaps in health literacy and to promote effective health literacy-informed interventions to reduce hospital pressure for low-acuity disease.

This study has some limitations. Dealing with patients discharged from PED, etiological agents were available for a limited number of patients, even with of the introduction of the antigenic tests for RSV in the middle of the epidemic season. Moreover, because of the aims of this paper, the characteristics of the patients admitted to OBI or pediatric wards were not considered. 

The high number of patients, the single center cohort, the real-world approach and the exclusion of SARS-CoV-2 as an etiological agent are the strength elements of this study.

## 5. Conclusions

A focus on discharged patients and PED revisits was needed, as only a few studies are available in the literature [31,32]. Our epidemiological observational study may be a suggestion for a further improvement of the management of these patients, as well as for the effective internal organization of pediatric departments. Moreover, it could be a starting point for perspective studies in collaboration with PCPs in order to improve out-of-hospital management of patients to reduce PED pressure during epidemic season.

Bronchiolitis surveillance is fundamental due to the epidemiological and clinical modifications observed during and after the SARS-CoV-2 pandemic in order to assess the economic and social impact of the disease not only on healthcare services but also on the community. 

Empowering the collaboration between all healthcare provisioners involved (in and out of hospital) is fundamental for the correct management of children, especially during epidemic seasons.

## Figures and Tables

**Table 1:** Total PED accesses, admissions and hospitalizations for bronchiolitis.

Epidemic Season	Total PED Accesses *n*	PED Accesses for Bronchiolitis *n* (% on Total Accesses)	Hospitalizations for Bronchiolitis *n* (% on Total Accesses)
2017–2018	18,369	273 (1.49)	174 (0.95)
2018–2019	18,300	314 (1.72)	178 (0.97)
2019–2020	16,776	266 (1.59)	132 (0.79)
2020–2021	10,301	18 (0.17)	32 (0.31)
2021–2022	16,182	419 (2.59)	244 (1.51)
2022–2023	18,401	512 (2.78)	265 (1.44)

**Table 2:** Characteristics of patients discharged home.

	Patients Discharged Home *n* = 284
Sex, *n* (%)	
Female	109 (38.4)
Male	175 (61.6)
Age, n (%)	
<12 months	278 (97.9)
≥12 months	6 (2.1)
Risk factors, *n* (%)	
SGA	10 (3.5)
Cardiovascular diseases	8 (2.8)
Respiratory complications with the need for perinatal ventilation	7 (2.5)
Kidney and Urinary Tract Disorders	5 (1.8)
ALTE	2 (0.7)
Other	12 (4.2)
Gestational age, *n* (%)	
<37 weeks	23/282 (8.1)
≥37 weeks	259/282 (91.8)
At least one sibling, *n* (%)	143/239 (59.8)
Nationality of parents	
Italian	182 (64.1)
Not Italian	102 (35.9)
Viral etiologies	
RSV-positive, *n* (%)	24/64 (37.5)
O2 Saturation, Median (min–max)	99 (93–100)
Respiratory Rate, Median (min–max)	45 (28–76)

SGA, Small for Gestational Age; ALTE, Apparent Life-Threatening Event.

**Table 3:** Clinical and demographic characteristics of PED revisits.

	PED Revisits *n* = 38
Sex, *n* (%)	
Female	19 (50)
Male	19 (50)
Age, *n* (%)	
≤12 months	38 (100)
≤6 months	32 (84.2)
Risk factors, *n* (%)	
Cardiovascular diseases	1 (2.6)
Gestational age, *n* (%)	
<37 weeks	3 (7.9)
≥37 weeks	35 (92.1)
At least one sibling, *n* (%)	23/34 (60.5)
Nationality of parents	
Italian	23 (60.5)
Not Italian	15 (39.5)
Viral etiologies	
RSV, n (%)	15/21 (71.4)
Co-infections, *n* (%)	3/9 (33.3)
O2 Saturation, Median (min-max) *	98 (86–100)
Respiratory Rate, Median (min-max) *	45 (28–68)

* *n* = 37.

## Data Availability

Data available upon request to the corresponding author. The data are not publicly available due to privacy reasons.

## Supplementary Materials

The following supporting information can be downloaded at: https://www.mdpi.com/article/10.3390/children11030268/s1, Table S1: diagnosis of bronchiolitis in PED in October 2022, detailed by triage code and outcome; Table S2: diagnosis of bronchiolitis in PED in November 2022, detailed by triage code and outcome; Table S3: diagnosis of bronchiolitis in PED in December 2022, detailed by triage code and outcome; Table S4: diagnosis of bronchiolitis in PED in January 2023, detailed by triage code and outcome; Table S5: diagnosis of bronchiolitis in PED in February 2023, detailed by triage code and outcome; Table S6: diagnosis of bronchiolitis in PED in March 2023, detailed by triage code and outcome.
