# Supplementary material for: Experience of an Italian Pediatric Third Level Emergency Department during the 2022–2023 Bronchiolitis Epidemic: A Focus on Discharged Patients and Revisits
Source: Children (Basel). 2024 Feb 21;11(3):268. doi: 10.3390/children11030268 (PMC10968752; doi:10.3390/children11030268)
Supplement: Supplementary file 1 [file children-11-00268-s001.zip › children-2870656-supplementary.pdf]

Supplementary materials

Table S1. Diagnosis of bronchiolitis in PED in October 2022, detailed by triage code and outcome.

	Code 5 n (%)	Code 4 n (%)	Code 3 n (%)	Code 2 n (%)	Code 1 n (%)
**Total**	0 (0)	4 (26,7)	8 (53,3)	3 (20)	0 (0)
Discharged home	0 (0)	3 (20)	5 (33,3)	2 (13,3)	0 (0)
Hospitalized	0 (0)	0 (0)	1 (6,7)	1 (6,7)	0 (0)
OBI	0 (0)	0 (0)	2 (13,3)	0 (0)	0 (0)
Clinic	0 (0)	0 (0)	0 (0)	0 (0)	0 (0)
Patient leaves before visit	0 (0)	0 (0)	0 (0)	0 (0)	0 (0)
Patient refuses hospitalization	0 (0)	1 (6,7)	0 (0)	0 (0)	0 (0)
Transferred to another hospital	0 (0)	0 (0)	0 (0)	0 (0)	0 (0)

Table S2. Diagnosis of bronchiolitis in PED in November 2022, detailed by triage code and outcome.

	Code 5 n (%)	Code 4 n (%)	Code 3 n (%)	Code 2 n (%)	Code 1 n (%)
**Total**	0 (0)	12 (10,6)	60 (53,1)	37 (32,7)	4 (3,5)
Discharged home	0 (0)	9 (8)	42 (37,2)	8 (7,1)	0 (0)
Hospitalized	0 (0)	0 (0)	5 (4,4)	14 (12,4)	3 (2,6)
OBI	0 (0)	3 (2,6)	13 (11,5)	15 (13,3)	1 (0,9)
Clinic	0 (0)	0 (0)	0 (0)	0 (0)	0 (0)
Patient leaves before visit	0 (0)	0 (0)	0 (0)	0 (0)	0 (0)
Patient refuses hospitalization	0 (0)	0 (0)	0 (0)	0 (0)	0 (0)
Transferred to another hospital	0 (0)	0 (0)	0 (0)	0 (0)	0 (0)

Table S3. Diagnosis of bronchiolitis in PED in December 2022, detailed by triage code and outcome.

	Code 5 n (%)	Code 4 n (%)	Code 3 n (%)	Code 2 n (%)	Code 1 n (%)
**Total**	0 (0)	26 (14,4)	81 (44,7)	66 (36,5)	8 (4,4)
Discharged home	0 (0)	24 (13,2)	43 (23,7)	24 (13,2)	0 (0)
Hospitalized	0 (0)	0 (0)	14 (7,7)	22 (12,1)	5 (2,8)
OBI	0 (0)	2 (1,1)	24 (13,2)	19 (10,4)	3 (1,6)
Clinic	0 (0)	0 (0)	0 (0)	0 (0)	0 (0)
Patient leaves before visit	0 (0)	0 (0)	0 (0)	1 (0,5)	0 (0)
Patient refuses hospitalization	0 (0)	0 (0)	0 (0)	0 (0)	0 (0)
Transferred to another hospital	0 (0)	0 (0)	0 (0)	0 (0)	0 (0)

Table S4. Diagnosis of bronchiolitis in PED in January 2023, detailed by triage code and outcome.

	Code 5<br>n (%)	Code 4<br>n (%)	Code 3<br>n (%)	Code 2<br>n (%)	Code 1<br>n (%)
**Total**	0 (0)	12 (11,3)	63 (59,5)	30 (28,3)	1 (0,9)
Discharged home	0 (0)	12 (11,3)	41 (38,7)	12 (11,3)	0 (0)
Hospitalized	0 (0)	0 (0)	8 (7,6)	12 (11,3)	1 (0,9)
OBI	0 (0)	0 (0)	14 (13,2)	6 (5,7)	0 (0)
Clinic	0 (0)	0 (0)	0 (0)	0 (0)	0 (0)
Patient leaves before visit	0 (0)	0 (0)	0 (0)	0 (0)	0 (0)
Patient refuses hospitalization	0 (0)	0 (0)	0 (0)	0 (0)	0 (0)
Transferred to another hospital	0 (0)	0 (0)	0 (0)	0 (0)	0 (0)

Table S5. Diagnosis of bronchiolitis in PED in February 2023, detailed by triage code and outcome.

	Code 5<br>n (%)	Code 4<br>n (%)	Code 3<br>n (%)	Code 2<br>n (%)	Code 1<br>n (%)
**Total**	0 (0)	8 (14,3)	35 (62,5)	13 (23,2)	0 (0)
Discharged home	0 (0)	7 (12,5)	24 (42,9)	5 (8,9)	0 (0)
Hospitalized	0 (0)	0 (0)	4 (7,1)	5 (8,9)	0 (0)
OBI	0 (0)	1 (1,8)	7 (12,5)	3 (5,4)	0 (0)
Clinic	0 (0)	0 (0)	0 (0)	0 (0)	0 (0)
Patient leaves before visit	0 (0)	0 (0)	0 (0)	0 (0)	0 (0)
Patient refuses hospitalization	0 (0)	0 (0)	0 (0)	0 (0)	0 (0)
Transferred to another hospital	0 (0)	0 (0)	0 (0)	0 (0)	0 (0)

Table S6. Diagnosis of bronchiolitis in PED in March 2023, detailed by triage code and outcome.

	Code 5<br>n (%)	Code 4<br>n (%)	Code 3<br>n (%)	Code 2<br>n (%)	Code 1<br>n (%)
**Total**	0 (0)	3 (7,3)	22 (53,7)	16 (39,0)	0 (0)
Discharged home	0 (0)	2 (4,9)	15 (36,6)	6 (14,6)	0 (0)
Hospitalized	0 (0)	0 (0)	3 (7,3)	7 (17,1)	0 (0)
OBI	0 (0)	1 (2,4)	4 (9,8)	3 (7,3)	0 (0)
Clinic	0 (0)	0 (0)	0 (0)	0 (0)	0 (0)
Patient leaves before visit	0 (0)	0 (0)	0 (0)	0 (0)	0 (0)
Patient refuses hospitalization	0 (0)	0 (0)	0 (0)	0 (0)	0 (0)
Transferred to another hospital	0 (0)	0 (0)	0 (0)	0 (0)	0 (0)
